# Arabidopsis OTU2 deubiquitinates cysteine protease RD21A to enhance clubroot resistance

**DOI:** 10.1111/tpj.70148

**Published:** 2025-04-14

**Authors:** Chao Li, Sha Li, Lu Feng, Jiasen Cheng, Jiatao Xie, Yang Lin, Yanping Fu, Kenichi Tsuda, Daohong Jiang, Tao Chen

**Affiliations:** ^1^ State Key Laboratory of Agricultural Microbiology Huazhong Agricultural University Wuhan China; ^2^ Hubei Key Laboratory of Plant Pathology, College of Plant Science and Technology Huazhong Agricultural University Wuhan China; ^3^ Hubei Hongshan Laboratory Wuhan China

**Keywords:** OTU2, deubiquitinating enzyme, clubroot, RD21A, plant resistance

## Abstract

Clubroot is a major threat to cruciferous crops worldwide, largely due to the complex pathogenesis of its causal agent, *Plasmodiophora brassicae*, and the limited availability of genetic resistance in plants. Previous research has shown that *P. brassicae* secretes the E3 ubiquitin ligase PbE3‐2, which targets and degrades the *Arabidopsis thaliana* cysteine protease RD21A to facilitate infection. In this study, we identified a plant defense mechanism that counteracts this pathogen virulence strategy. We found that the *A. thaliana* deubiquitinating enzyme OTU2, whose expression is upregulated during infection, interacts with RD21A. Notably, OTU2 stabilized RD21A by deubiquitination and inhibited the interaction between PbE3‐2 and RD21A. Furthermore, *OTU2* overexpression enhanced *A. thaliana* resistance to *P. brassicae* in an RD21A‐dependent manner. Collectively, our findings demonstrate that OTU2 deubiquitinates RD21A, protecting it from PbE3‐2‐mediated degradation and thereby mitigating *P. brassicae* virulence. This study provides new insights into plant immune mechanisms and offers potential strategies for developing clubroot‐resistant crops.

## Significance Statement

Protists secrete the ubiquitin ligase effector PbE3‐2 to target RD21A, thereby facilitating infection. In response, *Arabidopsis thaliana* employs the deubiquitinating enzyme OTU2 to counteract this effect by inhibiting the interaction. These findings highlight a dynamic arms race between *Plasmodiophora brassicae* and its host, driven by opposing ubiquitination and deubiquitination mechanisms. This work not only deepens our understanding of plant immune regulation but also identifies OTU2 as a potential target for improving resistance to clubroot.

## INTRODUCTION

*Plasmodiophora brassicae* is the causal agent of clubroot, a devastating disease affecting cruciferous plants and significantly reducing global agricultural production (Javed et al., 2023). Clubroot leads to abnormal root swelling and tumor‐like gall formation, resulting in severe yield losses or even complete crop failure (Hwang et al., 2012). The availability of genetic resistance to clubroot is limited. For instance, WeiTsing (Ochoa named RPB1), a small endoplasmic reticulum‐localized protein, functions as a calcium‐permeable cation‐selective channel and confers plant resistance to *P. brassicae* (Ochoa et al., 2023; Wang et al., 2023). Additionally, Gravot et al. (2024) have identified two adjacent Nucleotide binding and leucine‐rich repeat‐containing receptors (NLRs), *AT5G47260* and *AT5G47280*, that confer resistance to *P. brassicae*. Despite these advances, clubroot prevention and treatment are challenging due to the complex pathogenic mechanism of *P. brassicae*. During infection, *P. brassicae* secretes the E3 ubiquitin ligase PbE3‐2, which targets and degrades the host cysteine protease RD21A to suppress the plant immune response (Li et al., 2024).

Faced with constant threats from multiple pathogens, plants have evolved a sophisticated immune system to combat various pathogens. Pattern‐triggered immunity (PTI) and effector‐triggered immunity (ETI) function synergistically to confer resistance against pathogens (Yuan et al., 2021). To establish infection, pathogens secrete effectors that suppress plant immune responses. In turn, plants deploy intracellular NLRs to recognize these effectors and trigger a robust immune response, often accompanied by localized hypersensitive cell death (Zhou & Zhang, 2020). This highlights the importance of balancing immunity and growth when utilizing NLR genes in disease‐resistant breeding (Gao et al., 2024). Beyond NLR‐mediated recognition, plants also employ direct effector‐targeting strategies to attenuate pathogen virulence. For instance, *A. thaliana* lectin receptor‐like kinase LecRK‐IX.2 phosphorylates the bacterial effector AvrPtoB, reducing its virulence and enhancing plant immunity (Xu et al., 2020). This mechanism enables plants to mitigate pathogen virulence while minimizing the potential trade‐offs of excessive immune activation. However, whether plants utilize additional strategies to counteract pathogen effectors remains an open question.

Ubiquitination is a reversible post‐translational modification mediated by a cascade of enzymes, including ubiquitin‐activating enzyme (E1), ubiquitin‐conjugating enzyme (E2) and ubiquitin ligase (E3), which sequentially attach ubiquitin to target proteins (Smalle & Vierstra, 2004). The reverse process, deubiquitination, is carried out by deubiquitinating enzymes (DUBs), which cleave ubiquitin from substrates, thereby modulating ubiquitination levels (Lange et al., 2022). Together, ubiquitination and deubiquitination regulate protein homeostasis and are essential for diverse biological processes. In plants, DUBs play critical roles in growth, development and immunity. For example, in rice, PICI1 deubiquitinates the methionine synthase OsMETS1, enhancing its stability and contributing to immune regulation (Zhai et al., 2022). In *A. thaliana*, the homologous DUBs UBP12 and UBP13 interact with NPR3 in the nucleus in a salicylic acid (SA)‐dependent manner, removing polyubiquitin chains to prevent NPR3 degradation, thereby negatively regulating immunity (Zhou et al., 2023). Additionally, UBP12 and UBP13 target the brassinosteroid receptor BRASSINOSTEROID INSENSITIVE1 (BRI1), removing K‐63‐linked ubiquitin chains to regulate its degradation (Luo et al., 2022). The DUBs also modulate the jasmonic acid (JA) signaling pathway by stabilizing the transcription factor MYC2 through deubiquitination, preventing its degradation by the 26S proteasome (Jeong et al., 2017). *A. thaliana* OTU1 functions as a histone DUB involved in the transcriptional repression of *DA1* and *DA2*, genes that control organ and seed size (Keren et al., 2020). Despite these advances, the functions of many *A. thaliana* DUBs remain largely unexplored, particularly their interactions with E3 ubiquitin ligases in the regulation of specific protein targets.

Papain‐like cysteine proteases (PLCPs) are essential components of plant immunity against a broad spectrum of pathogens (Misas‐Villamil et al., 2016). RESPONSIVE TO DEHYDRATION 21A (RD21A), a PLCP, contributes to resistance against *Botrytis cinerea*, *Pseudomonas syringae* and *P. brassicae* (Li et al., 2024; Liu et al., 2020; Shindo et al., 2012). To overcome plant defenses, pathogens deploy effectors that target PLCPs, suppressing immune responses. As a key immune‐associated PLCP, RD21A is a common target of diverse pathogens. The *Xanthomonas* effector AvrRxo1 enhances the activity of the *A. thaliana* E3 ubiquitin ligase SINAT4, promoting RD21A degradation (Liu et al., 2020). *P. brassicae* secretes its own E3 ubiquitin ligase to facilitate RD21A degradation (Li et al., 2024). Additionally, the root‐knot nematode effector MiCE108 targets and degrades RD21A, suppressing plant defense and promoting parasitism (Yu et al., 2024). These findings suggest that pathogen effectors degrade RD21A or enhance its degradation via the ubiquitination pathway to increase virulence. Beyond pathogen‐mediated degradation, RD21A is subject to regulation by the ubiquitination pathway (Kim & Kim, 2013; Li et al., 2024). However, whether RD21A is modulated through deubiquitination and how plants counteract its pathogen‐induced degradation remain unclear. Investigating the regulatory mechanism of RD21A in disease resistance is of great significance for developing disease‐resistant crops.

In this study, we identified an *A. thaliana* DUB, ovarian tumor domain (OTU)‐containing deubiquitilating enzyme 2 (OTU2) as an interactor of the *P. brassicae* effector PbE3‐2, based on our previously published co‐immunoprecipitation data. We found that OTU2 directly interacts with RD21A – the target of PbE3‐2 – and inhibits the interaction between PbE3‐2 and RD21A. OTU2 deubiquitinates RD21A, preventing its degradation via the 26S proteasome pathway. Notably, *OTU2* overexpression enhances resistance to *P. brassicae* in an RD21A‐dependent manner. These findings elucidate a mechanism by which plants safeguard the key immune protease RD21A from effector‐mediated degradation, advancing our understanding of plant counter‐defense strategies against pathogen effectors.

## RESULTS

### RD21A overexpression mitigates PbE3‐2‐mediated virulence

**Figure 1:**
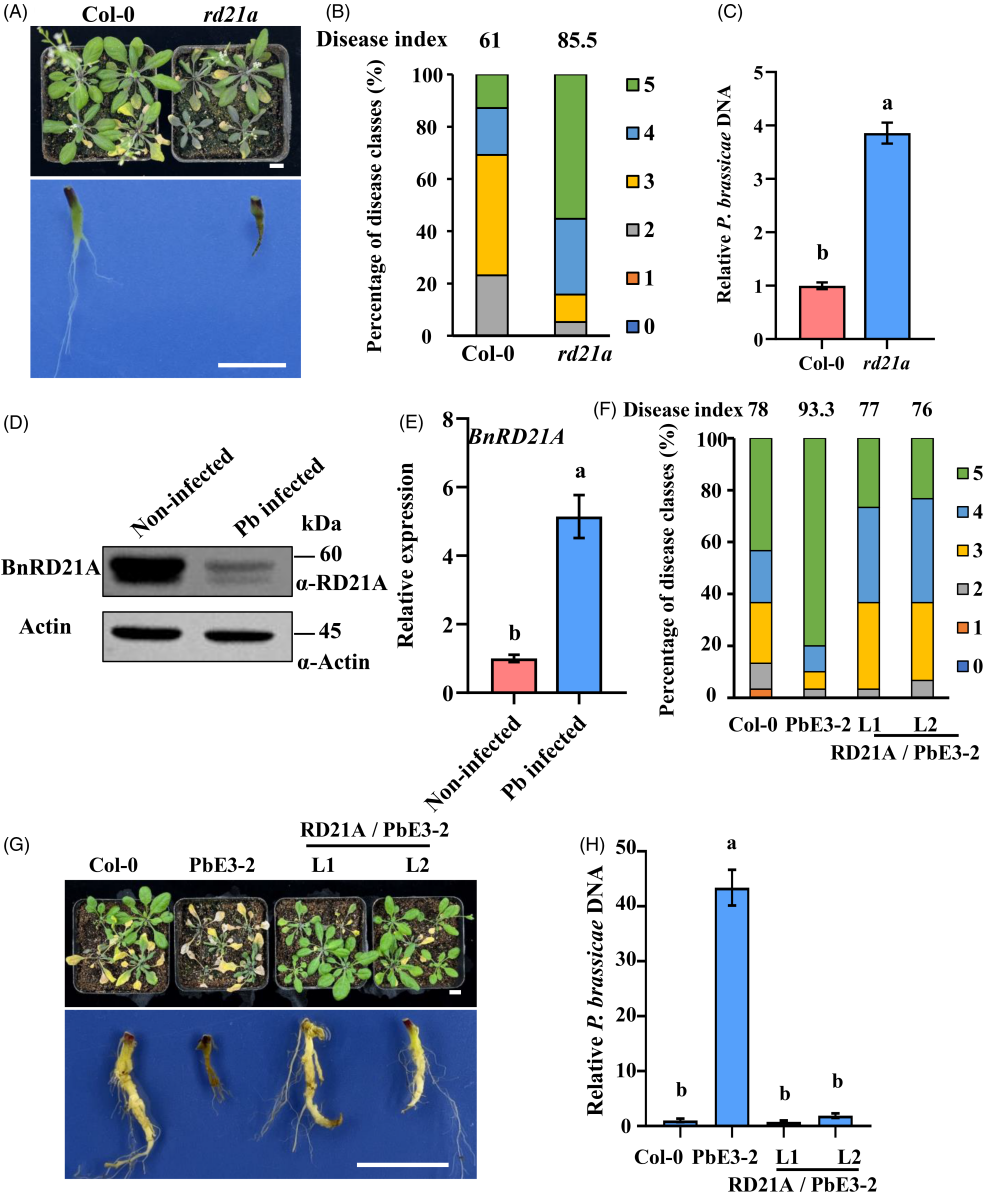
RD21A overexpression counteracted PbE3‐2‐mediated virulence. (A) Phenotype of Col‐0 and *rd21a* at 21 days after *Plasmodiophora brassicae* inoculation. Bars: 1 cm. (B) Disease index of Col‐0 and *rd21a*. The percentages of plants in the individual disease classes are shown; Col‐0 and *rd21a* were analyzed using 39 and 38 plants, respectively. (C) Relative pathogen DNA titer (*PbACTIN* /*AtACTIN2*) 21 dpi, n = 3 biological replicates, data are shown as mean ± SD, different letters indicate statistically significant differences (Student's *t*‐test, two‐tailed). (D) *Brassica nap*us BnRD21A protein level with or without infection by *P. brassicae*. The representative roots from three plants with or without infection by *P. brassicae* were used to extract total protein. (E) *B. nap*us BnRD21A transcriptional level with or without infection by *P. brassicae*. *n* = 3. A significant difference was determined by the Student's *t*‐test (two‐tailed). Different letters (a, b) indicate significant differences. Data are shown as mean ± SD. (F) Disease index of Col‐0, PbE3‐2 and RD21A/PbE3‐2 overexpression plants. The percentages of plants in the individual disease classes are shown. Each line used 30 plants for analysis. (G) Phenotype of Col‐0, PbE3‐2 and RD21A/PbE3‐2 overexpression plants at 21 days after *P. brassica*e inoculation. Bars: 1 cm. (H) Relative pathogen DNA titer (*PbACTIN* /*AtACTIN2*) 21 dpi, *n* = 3 biological replicates, data are shown as mean ± SD, statistical analysis was performed by one‐way anova (significance set at *P* ≤ 0.05). Different letters (a, b) indicate significant differences.

RD21A, a member of the PLCP family, plays a central role not only in both plant drought tolerance and plant resistance against pathogens. To investigate its function in resistance against pathogens, we assessed the resistance of *A. thaliana rd21a* mutant against *P. brassicae*. After 21 days post‐inoculation, *rd21a* mutant plants exhibited severe disease symptoms, including extensive blackening and wilting of the aboveground tissues, as well as severe root rot, which impaired water and nutrient uptake, ultimately leading to plant death (Figure 1A). Disease severity assessment revealed that over 50% of *rd21a* mutant plants reached the highest disease index (class 5), whereas most Col‐0 plants displayed moderate disease severity, with disease indices primarily in classes 3 and 4 (Figure 1B). Consistently, qPCR analysis of *P. brassicae* biomass in infected roots revealed a significantly higher pathogen load in *rd21a* mutants compared to Col‐0 plants (Figure 1C).

We previously reported that *P. brassicae* secretes the E3 ubiquitin ligase PbE3‐2, which degrades RD21A, thereby reducing *A. thaliana* resistance to *P. brassicae* (Li et al., 2024). To further investigate this mechanism, we analyzed the protein and mRNA levels of RD21A in rapeseed plants during *P. brassicae* invasion. Due to the small size of Arabidopsis roots, we examined RD21A protein levels in *Brassica napus* roots following *P. brassicae* invasion. As expected, BnRD21A protein levels were substantially reduced upon infection (Figure 1D), while its mRNA levels increased (Figure 1F), suggesting post‐transcriptional regulation. To test whether high RD21A abundance could counteract PbE3‐2‐mediated degradation, we overexpressed *RD21A‐FLAG* in *PbE3‐2‐GFP* transgenic *A. thaliana* and generated L1 and L2 overexpression lines (Figure S1a). At 21 days post‐inoculation, *P. brassicae* susceptibility was significantly higher in PbE3‐2‐GFP transgenic plants compared to Col‐0 (Figure 1F,G). However, L1 and L2 showed disease susceptibility levels similar to Col‐0. Furthermore, while *P. brassicae* biomass was elevated in PbE3‐2‐GFP transgenic plants compared to Col‐0, it remained comparable to Col‐0 in L1 and L2 lines (Figure 1H). These findings indicate that RD21A overexpression mitigates PbE3‐2‐mediated RD21A degradation, thereby counteracting its virulence function.

### *A. thaliana* deubiquitinating enzyme OTU2 interacts with RD21A

**Figure 2:**
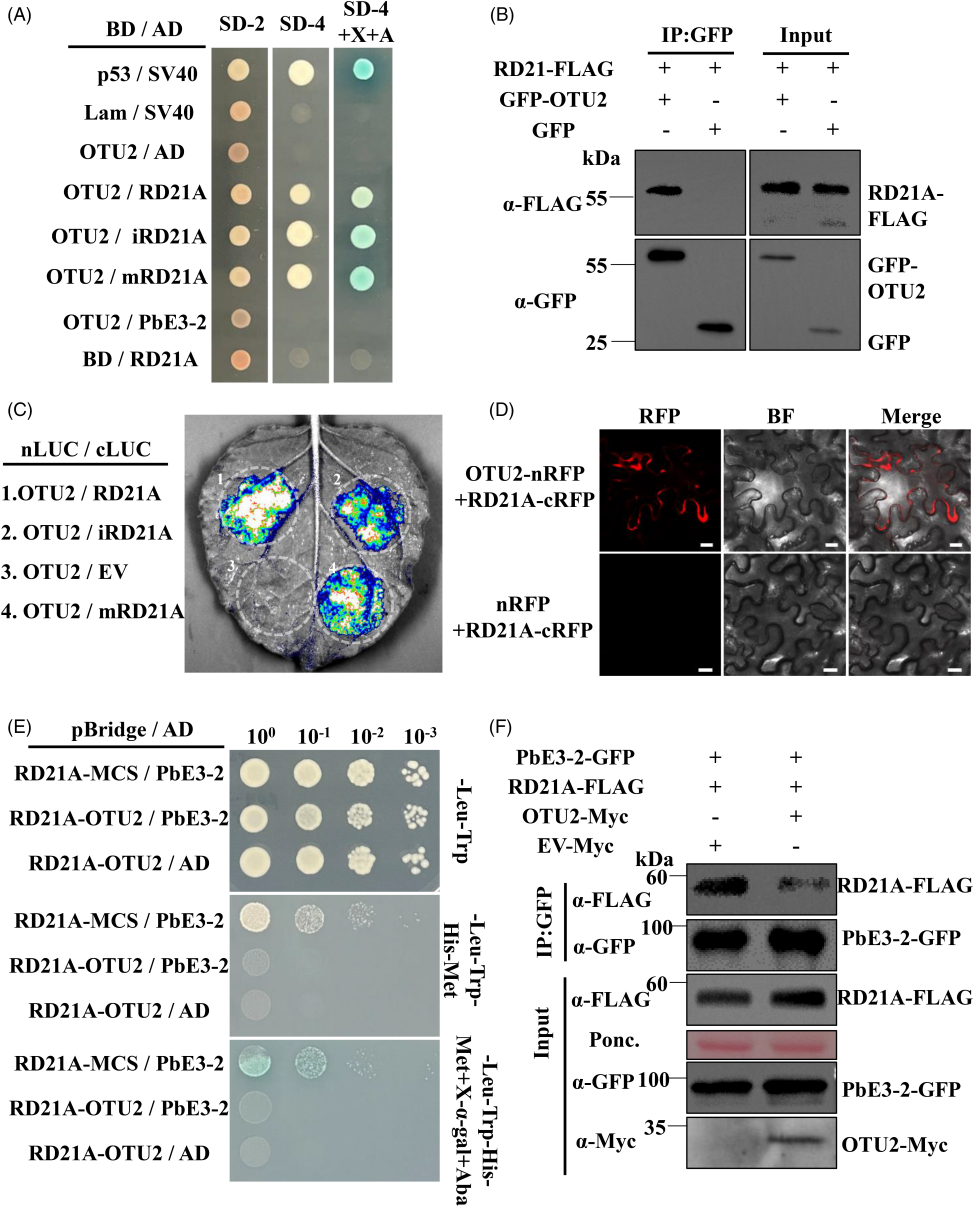
OTU2 interacts with RD21A and inhibits the interaction between PbE3‐2 and RD21A. (A) Y2H assays show that OTU2 interacts with RD21A, iRD21A and mRD21A but not with PbE3‐2 BD, indicating a pGBKT7 vector. AD indicates the pGADT7 vector. p53/SV40 is a positive control; Lam/SV40 is a negative control. SD−Leu−Trp medium (SD‐2) was used for screening clones harboring both plasmids and SD−Leu–Trp–His–Ade medium (SD‐4) with/without X‐α‐Gal (X) and aureobasidin A. (B) Co‐IP confirmed that OTU2 interacts with RD21A *in vivo*. IP was performed with α‐GFP agarose beads, followed by the detection of RD21A with α‐FLAG antibody. Input and IP proteins were immunoblotted with α‐FLAG and α‐GFP antibodies. (C) Split‐LUC assay confirmed that OTU2 interacts with RD21, iRD21A and mRD21A in plants. OTU2/EV was used as a negative control. (D) BiFC confirmed the interaction between OTU2 and RD21A. OTU2 is fused to the N‐terminus of RFP; RD21A is fused to the C‐terminus of RFP. The corresponding GV3101 strains carrying the target plasmid are transiently expressed in the *N. benthamiana* and observed with a confocal laser‐scanning microscope. Bars: 20 μm. (E) Y3H assay showed that OTU2 inhibited the interaction between PbE3‐2 and RD21A. Interaction of PbE3‐2 with RD21A was verified on −Leu−Trp−His−Met+X‐α‐gal+AbA medium when OTU2 was present or absent. pBridge‐RD21A‐OTU2 + AD served as a negative control. (F) Co‐IP assay showed that OTU2 inhibited the interaction between PbE3‐2 and RD21A. IP was performed with α‐GFP agarose beads, followed by the detection of RD21 with α‐FLAG antibody. Input was immunoblotted with α‐FLAG, α‐GFP and α‐Myc antibodies. Protein loading was shown by Ponceau S staining of Rubisco.

Our previous study has demonstrated that the *P. brassicae* ubiquitin ligase PbE3‐2 interacts with RD21A. To further explore this interaction network, we revisited our immunoprecipitation data for PbE3‐2 and identified ovarian tumor domain (OTU)‐containing deubiquitilating enzyme 2 (OTU2) as a potential interactor (Table S2). Given that OTU2 functions as a DUB, we hypothesized that it might deubiquitinate RD21A within the protein complex, thereby protecting RD21A from PbE3‐2‐mediated degradation. To investigate this, we performed a yeast two‐hybrid (Y2H) assay, which confirmed that OTU2 interacts with RD21A but not with PbE3‐2 (Figure 2A; Figure S1b). This suggests that OTU2 was co‐immunoprecipitated with PbE3‐2 through its interaction with RD21A, rather than directly binding to PbE3‐2. Consistent with these results, Split‐LUC assays demonstrated that OTU2 interacts with full‐length RD21A (RD21A), intermediate RD21A (iRD21A) and mature RD21A (mRD21A) (Figure 2C). To further validate the OTU2–RD21A interaction, we performed a co‐immunoprecipitation (Co‐IP) assay. When GFP‐tagged OTU2 or GFP was co‐expressed with FLAG‐tagged RD21A in *Nicotiana benthamiana* leaves, RD21A‐FLAG was successfully co‐immunoprecipitated with GFP‐OTU2 but not with GFP alone (Figure 2B). Additionally, BiFC assays demonstrated that OTU2 interacts with RD21A in both the cytoplasm and the cell membrane (Figure 2D; Figure S1c). Together, these results confirm that OTU2 interacts with RD21A, highlighting its potential role in counteracting the PbE3‐2‐mediated degradation of RD21A.

OTU2 is one of the 12 members of the *A. thaliana* OTU family (Figure S2a). While OTU2 is known to hydrolyze K48‐ and K63‐linked ubiquitin chains *in vitro* (Radjacommare et al., 2014), its specific function in plants remains largely unexplored. OTU2 contains an OTU domain (11–123 aa) at its N‐terminus and a C2H2 zinc finger domain at its C‐terminus (179–201 aa) (Figure S2b). To investigate the structural requirements for the interaction between OTU2 and RD21A, we used truncated derivatives of OTU2 in Y2H assays. We found that RD21A interacts with OTUc (124–208 aa) but not with OTUn (11–123 aa), indicating that the C2H2 zinc finger domain is essential for the interaction between OTU2 and RD21A (Figure S2c).

### OTU2 inhibits the interaction between PbE3‐2 and RD21A

Given that PbE3‐2 ubiquitinates RD21A and OTU2 interacts with RD21A, we hypothesized that OTU2 may interfere with the interaction between PbE3‐2 and RD21A. The Y3H experiment results supported this hypothesis, showing that OTU2 blocks the interaction between PbE3‐2 and RD21A. As shown in Figure 2(E), yeast colonies carrying the PbE3‐2 and RD21A constructs grew on the defective medium, whereas colonies with an additional OTU2 construct did not, indicating that OTU2 disrupts this interaction. In Co‐IP assays, RD21A‐FLAG and PbE3‐2‐GFP were co‐expressed with either OTU2‐Myc or Ev‐Myc in *N. benthamiana* leaves. IP was then performed using α‐GFP agarose beads. Notably, the presence of OTU2‐Myc suppressed the co‐precipitation of RD21A‐FLAG with PbE3‐2‐GFP, suggesting that OTU2 interferes with the interaction between PbE3‐2 and RD21A in plants (Figure 2F).

### OTU2 deubiquitinates RD21A and increases its stability

**Figure 3:**
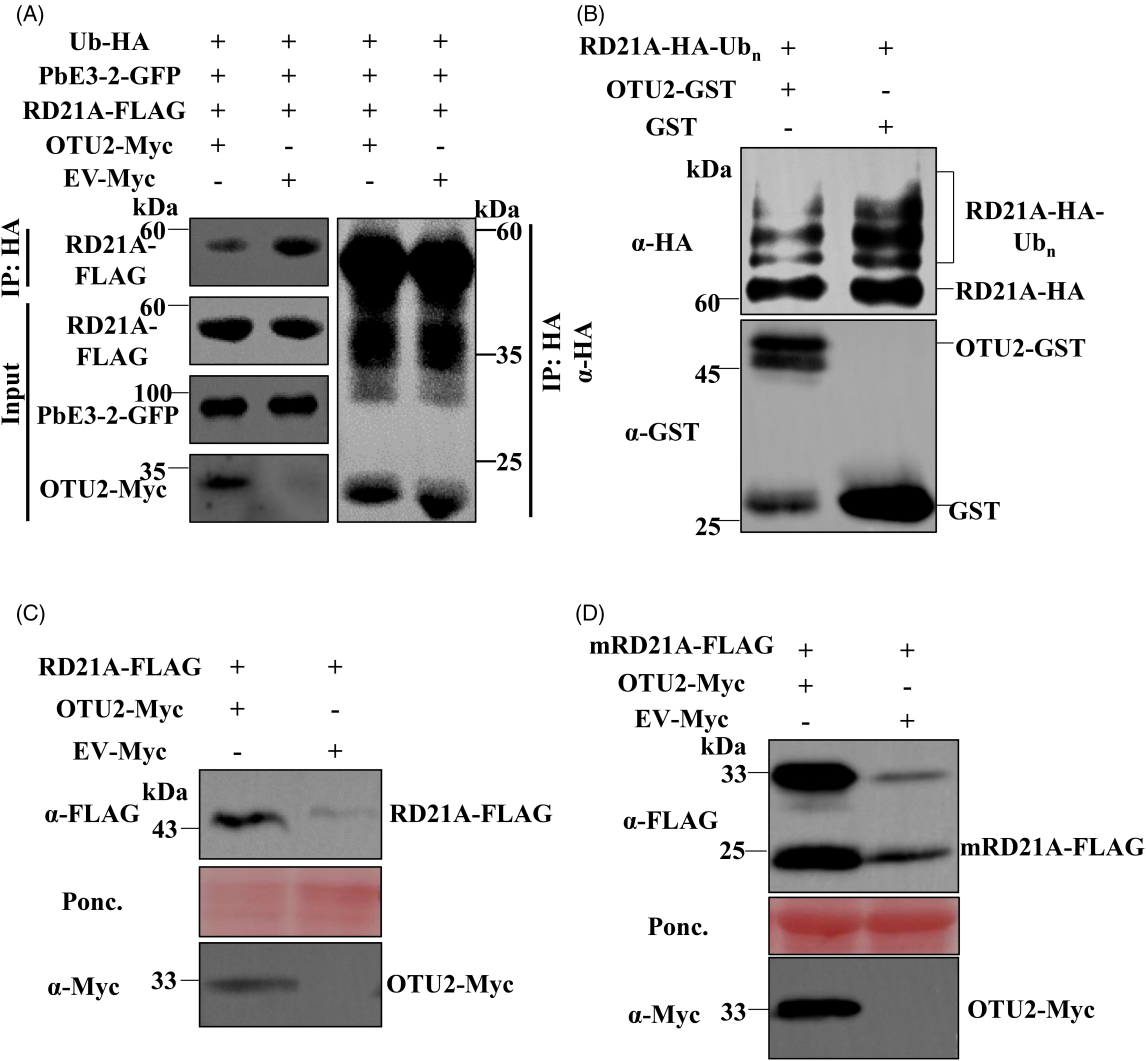
OTU2 deubiquitinates RD21A and increases its protein stability. (A) OTU2 deubiquitinates RD21A *in vivo*. IP was performed with α‐HA agarose beads, followed by the detection of RD21A with α‐FLAG antibody. Input proteins were immunoblotted with α‐FLAG, α‐GFP and α‐Myc antibodies. (B) OTU2 deubiquitinates RD21A *in vitro*. RD21A‐HA‐Ubn ubiquitinated by PbE3‐2 was purified according to Li et al. (2024) and incubated with OTU2‐GST or GST, respectively. RD21A‐HA‐Ubn was subsequently detected by immunoblotting with α‐HA antibody. (C) OTU2 promotes the stability of RD21A. *Agrobacterium* harboring RD21A‐FLAG and OTU2‐Myc or Ev‐Myc constructs were coinfiltrated in *N. benthamiana* leaves. Immunoblotting was performed with α‐FLAG and α‐Myc antibodies against RD21A and OTU2‐Myc, respectively. Protein loading was shown by Ponceau S staining of Rubisco. (D) OTU2 promotes the stability of mRD21A. *Agrobacterium* harboring mRD21A‐FLAG and OTU2‐Myc or Ev‐Myc constructs were coinfiltrated in *N. benthamiana* leaves. Immunoblotting was performed with α‐FLAG and α‐Myc antibodies against mRD21A and OTU2‐Myc, respectively. Protein loading was shown by Ponceau S staining of Rubisco.

Given that OTU2 is a DUB, we hypothesized that it may remove ubiquitin modifications from RD21A. To test this, Ub‐HA, PbE3‐2‐GFP and RD21A‐FLAG were co‐expressed with either OTU2‐Myc or Ev‐Myc in *N. benthamiana* leaves. To prevent the degradation of RD21A‐FLAG, the leaves were also infiltrated with 50 μm MG132. IP was performed using anti‐HA beads, and the ubiquitination level of RD21A‐FLAG was determined by quantifying the protein precipitated with Ub‐HA. The results showed that, compared with the control, the presence of OTU2 decreased the amount of RD21A‐FLAG labeled with Ub‐HA, suggesting that OTU2 deubiquitinates RD21A (Figure 3A). *In vitro* assays further supported these findings. RD21A‐HA‐Ubn, ubiquitinated by PbE3‐2 in *Escherichia coli* using a previously established protocol, was incubated with GST‐OTU2. The ubiquitination level of RD21A‐HA‐Ubn was markedly reduced following incubation with GST‐OTU2, whereas no reduction was observed with GST alone. Transient expression of PbE3‐2‐GFP, RD21A‐FLAG and OTU2‐Myc in *N. benthamiana* revealed that OTU2 increased RD21A accumulation compared to the control with an empty vector (Figure S3a), suggesting that OTU2 may protect RD21A from degradation mediated by PbE3‐2. Furthermore, we examined whether OTU2 similarly protects RD21A from degradation by the *Meloidogyne incognita* effector MiCE108 (Yu et al., 2024). Agrobacterium‐mediated transient expression in *N. benthamiana* confirmed that OTU2 can also safeguard RD21A from MiCE108‐mediated degradation (Figure S3b). These results collectively suggest that OTU2 effectively removes ubiquitin from RD21A, counteracting the ubiquitination induced by PbE3‐2.

To further elucidate the role of OTU2 in stabilizing RD21A, we co‐expressed RD21A‐FLAG with OTU2‐Myc or an empty vector in *N. benthamiana* leaves. RD21A‐FLAG exhibited increased accumulation when co‐expressed with OTU2‐Myc compared to the control (Figure 3C). Similarly, mRD21A also showed enhanced protein accumulation in the presence of OTU2‐Myc (Figure 3D). To determine whether OTU2 increases RD21A protein accumulation in *A. thaliana*, we performed immunoblot analysis using an anti‐RD21A antibody. As expected, transgenic *A. thaliana* overexpressing *OTU2* displayed significantly higher mRD21A levels than Col‐0 and *otu2* mutant plants (Figure S4e). Together, these results suggest that OTU2 increases RD21A protein accumulation by deubiquitinating RD21A, thereby preventing its degradation via the 26S proteasome.

### *A. thaliana OTU2* overexpression plants exhibit enhanced pathogen resistance

**Figure 4:**
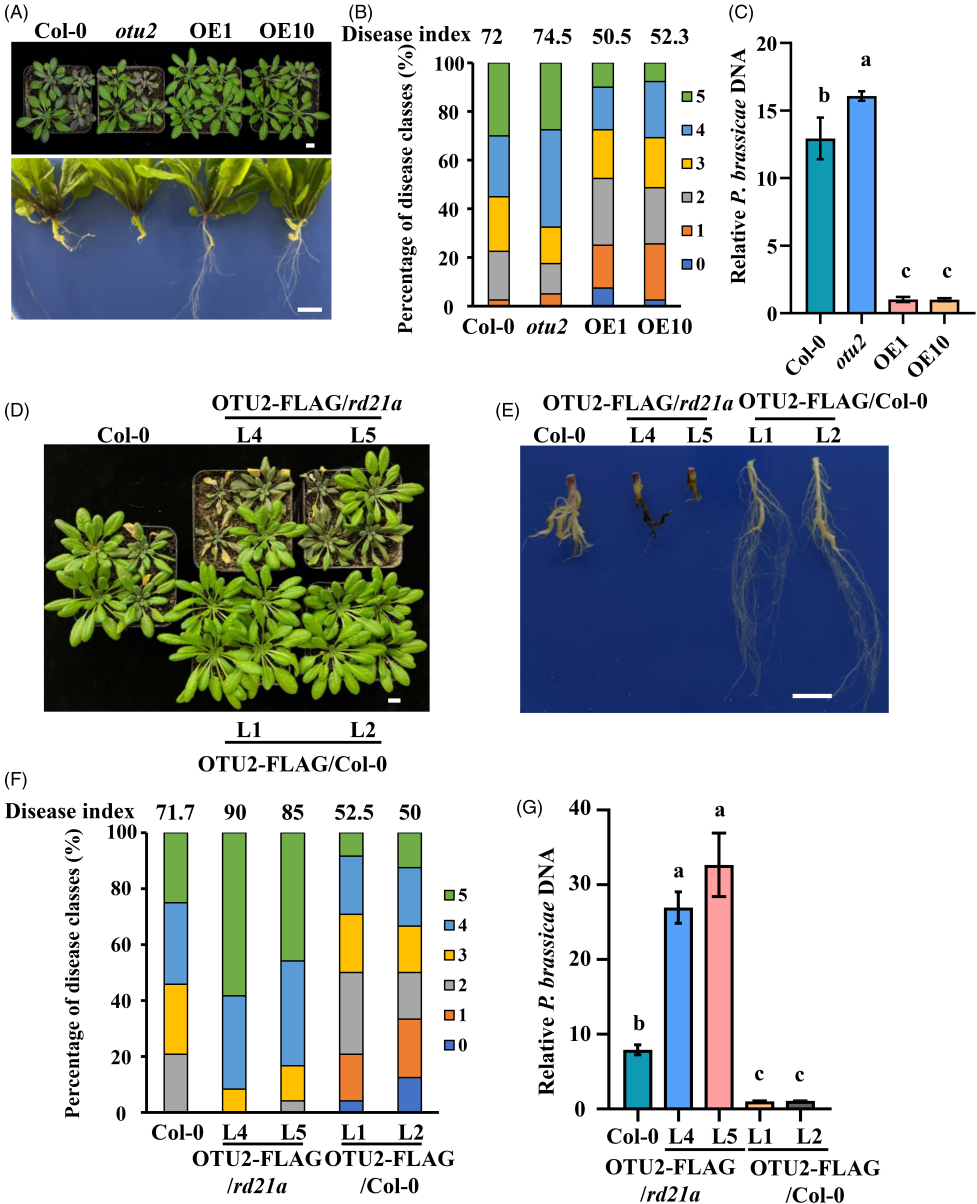
OTU2 promoted Arabidopsis resistance to clubroot in an RD21A‐dependent manner. (A) Phenotype of Col‐0, *otu2*, *OTU2* overexpression lines OE1 and OE10 at 21 days after *Plasmodiophora brassicae* inoculation. Bars: 1 cm. (B) Disease index of Col‐0, *otu2* and *OTU2* overexpression lines. The percentages of plants in the individual disease classes are shown; Col‐0, *otu2* and OE1 were analyzed using 40 plants, and OE10 was analyzed using 39 plants. (C) Relative pathogen DNA titer (*PbACTIN*/*AtACTIN2*) 21 dpi, *n* = 3 biological replicates. (D, E) Aboveground (D) and root (E) phenotype of Col‐0, OTU2‐FLAG overexpression lines L4, L5 (*rd21a* background) and L1, L2 (Col‐0 background) at 21 days after *P. brassicae* inoculation. Bars: 1 cm. (F) Disease index of Col‐0 and OTU2‐FLAG overexpression lines. The percentages of plants in the individual disease classes are shown. Each line was analyzed using 24 plants. (G) Relative pathogen DNA titer (*PbACTIN* /*AtACTIN2*) 21 dpi, *n* = 3 biological replicates. Statistical analyses (C, G) were performed by one‐way anova (significance set at *P* ≤ 0.05). Different letters (a–c) indicate significant differences.

Considering that OTU2 increases RD21A accumulation, we hypothesized that *OTU2* overexpression increases plant resistance against pathogens. To test this, we generated A. *thaliana* transgenic lines constitutively expressing *OTU2* under the control of the CaMV 35S promoter in Col‐0 (Figure S4). To evaluate their pathogen resistance, we challenged *OTU2* overexpression and Col‐0 plants with *P. brassicae* and observed their phenotypes at 21 days post‐inoculation (dpi) (Figure 4A). In Col‐0 and *otu2* mutant plants, typical galls formed on the primary roots and lateral roots were severely damaged following *P. brassicae* infection. In contrast, *OTU2*‐overexpressing plants exhibited fewer galls on the primary roots and retained more intact lateral roots. Moreover, the aboveground portions of Col‐0 and *otu2* plants showed symptoms of yellowing, wilting and premature senescence, whereas *OTU2*‐overexpressing plants exhibited fewer disease symptoms (Figure 4A,B). qPCR analysis of *P. brassicae* biomass further confirmed reduced pathogen accumulation in OE1 and OE10 lines compared to Col‐0 and *otu2* plants (Figure 4C). These results indicate that *OTU2* overexpression enhances *A. thaliana* resistance to *P. brassicae*, likely by increasing RD21A accumulation.

### OTU2‐mediated resistance enhancement requires RD21A

To determine whether the resistance enhancement conferred by OTU2 depends on RD21A, we generated OTU2‐overexpressing lines in the *rd21a* mutant background. Immunoblot analysis confirmed the successful overexpression of OTU2‐FLAG in both *rd21a* (lines L4 and L5) and Col‐0 (lines L1 and L2) backgrounds (Figure S5a). We then assessed the resistance of these lines to *P. brassicae*. Notably, L4 and L5 (*rd21a* background) exhibited more severe disease symptoms than Col‐0, with nearly complete necrosis of the aboveground tissues and extensive root rot and blackening (Figure 4D–F). In contrast, L1 and L2 (Col‐0 background) displayed increased resistance, with minimal disease symptoms in the aerial parts and only slight swelling of the primary roots (Figure 4D–F). qPCR analysis of the *P. brassicae* biomass corroborated these observations. Compared to Col‐0, pathogen biomass was significantly higher in L4 and L5 but markedly lower in L1 and L2 (Figure 4G). To determine whether OTU2 protects RD21A from degradation during *P. brassicae* infection, we analyzed RD21A protein levels in the roots of *A. thaliana* at 2 dpi. Compared to Col‐0 and *otu2* mutant plants, RD21A accumulation was substantially higher in the roots of OTU2‐overexpressing plants (Figure S5b). These findings indicate that OTU2‐mediated resistance enhancement is dependent on the presence of RD21A, suggesting that OTU2 exerts its protective effects through the stabilization of RD21A during pathogen infection.

## DISCUSSION

We propose a model in which OTU2 regulates plant immunity by stabilizing RD21A (Figure S6). During *P. brassicae* infection, the pathogen secretes the E3 ubiquitin ligase PbE3‐2, which targets RD21A for ubiquitination and degradation, thereby suppressing plant immunity. In response, *A. thaliana* employs the deubiquitinating enzyme OTU2 to counteract this effect by inhibiting the interaction between PbE3‐2 and RD21A. Furthermore, OTU2 deubiquitinates RD21A, promoting its accumulation and thereby enhancing *A. thaliana* resistance to *P. brassicae*. These findings highlight a dynamic arms race between *P. brassicae* and its host, driven by opposing ubiquitination and deubiquitination mechanisms. This work not only deepens our understanding of plant immune regulation but also identifies OTU2 as a potential target for improving resistance to clubroot disease in crop breeding and disease management strategies.

Pathogens employ diverse strategies to suppress plant immunity, including the secretion of effectors that manipulate host cellular processes. A well‐documented mechanism involves pathogen‐secreted effectors with E3 ubiquitin ligase activity, which target key plant immune components for ubiquitination and subsequent degradation. For example, *P. brassicae* secretes PbE3‐2 that ubiquitinates the *A. thaliana* RD21A, leading to its degradation and thereby weakening the host immune response (Li et al., 2024). Similarly, *P. syringae* AvrPtoB, an effector with E3 ligase activity, ubiquitinates multiple plant immune‐related proteins, including the exocyst subunit EXO70B1, the flagellin receptor FLS2, the LysM receptor kinase CERK1 and the immune regulator NPR1, to disrupt plant defense responses (Chen et al., 2017; Gimenez‐Ibanez et al., 2009; Göhre et al., 2008; Wang et al., 2019). However, how plants counteract these ubiquitination‐mediated virulence strategies remains largely unknown. While pathogens have evolved E3 ubiquitin ligases to target and degrade key immune components, the extent to which plants employ deubiquitination mechanisms to stabilize these proteins and maintain immune responses is not well understood.

We have demonstrated that OTU2 counteracts PbE3‐2‐mediated RD21A degradation by deubiquitinating RD21A, a member of the PLCP family. RD21A plays a critical role in plant immunity and contributes to resistance against a broad range of pathogens, including fungi, bacteria, nematodes and *P. brassicae* (Li et al., 2024; Liu et al., 2020; Pogorelko et al., 2019; Shindo et al., 2012). Given its essential function in plant defense, the stability of RD21A must be tightly regulated. It has been shown that plants regulate the stability of RD21A through E3 ubiquitin ligases. For instance, the *A. thaliana* ubiquitin ligase AtAIRP3/LOG2 ubiquitinates RD21A and SINAT4 promotes its degradation via a proteasome‐dependent pathway (Kim & Kim, 2013; Liu et al., 2020). Given that pathogen effectors also target RD21A for degradation via ubiquitination (Li et al., 2024; Liu et al., 2020), it is plausible that plants have evolved a counteracting mechanism to protect RD21A from pathogen‐induced degradation. Indeed, we have shown that OTU2 inhibits the interaction between PbE3‐2 and RD21A. Furthermore, OTU2 deubiquitinates RD21A both *in vitro* and *in vivo*, thereby increasing RD21A stability. Thus, OTU2‐mediated RD21A deubiquitination serves as a novel mechanism for counteracting *P. brassicae* infection and could be explored as a strategy for clubroot disease management.

Our experiments confirmed that OTU2 functions as a deubiquitinating enzyme in response to *P. brassicae* PbE3‐2 and holds significant potential for application in disease‐resistant breeding. However, several key questions remain unanswered. It is still unclear which E3 ubiquitin ligases work in conjunction with OTU2 to regulate RD21A homeostasis under normal physiological conditions. Further experiments are required to elucidate how OTU2 modulates RD21A stability in the absence of pathogen invasion and whether additional regulatory factors are involved in this process. Moreover, the number of DUBs in plants is considerably smaller than that of E3 ubiquitin ligases, implying that individual DUBs may regulate multiple substrates targeted by different E3 ligases. Therefore, it is essential to explore additional substrates of OTU2 to gain a comprehensive understanding of its biological functions. Investigating the broader role of OTU2 in plant immunity and proteostasis will provide valuable insights into its potential applications in crop improvement strategies.

## MATERIALS AND METHODS

### Plants and growth conditions

Arabidopsis and *N. benthamiana* plants were grown in a greenhouse at 12 h day, 12 h night, 22°C and 60% relative humidity. *OTU2* overexpression lines were generated by using *Agrobacterium* (*A. tumefaciens*)‐mediated transformation by floral dipping. The T‐DNA insertion mutant *otu2* (SALK_089249) was obtained from the Nottingham *A. thaliana* Stock Centre (NASC).

### Pathogen inoculation assays

*P. brassicae* inoculation was performed as previously described (Li et al., 2024). One milliliter of 1.0 × 10^6^ spores ml^−1^ *P. brassicae* resting spore suspension was inoculated onto 2‐week‐old *A. thaliana* seedlings through the soil around the roots, and disease analysis was carried out at 21 dpi. For each experiment, 24 to 40 plants were assessed. The experiment was repeated three times with similar results.

### Measurement of *P. brassicae* biomass

Roots of *P. brassicae*‐infected plants were harvested at 21 dpi, and DNA was extracted using the cetyltrimethylammonium bromide method. qPCR was performed in a CFX96 thermal cycler (Bio‐Rad); system software was used to calculate Cp values according to the second derivative max method for host target *A. thaliana ACTIN2* gene (AT3G18780) and pathogen target *P. brassica ACTIN* gene (AY452179.1). Primer sequences are listed in Table S1. The pathogen DNA titer relative to the host was calculated from the difference between CtPbACTIN and CtChina Agriculture Research System (CARS‐12)AtACTIN2. Three representative roots of each sample were selected for detection; three technical replicates were performed for each gene for each sample and were averaged. The experiment was repeated three times with similar results.

### RNA isolation and RT‐qPCR analysis

Total RNA was isolated from rapeseed roots inoculated with *P. brassica* for 30 days with a Trizol reagent (Invitrogen; Cat.15596026, Carlsbad, CA, USA). One microgram RNA was reverse transcribed to synthesize first‐strand cDNA at 50°C for 30 min (Vazyme; Cat. R223‐01, Nanjing, China) and reverse transcription‐quantitative RT‐qPCR analysis was performed with SYBR green Supermix (Bio‐Rad; Cat. 1725124, Hercules, CA, USA) on the Bio‐Rad CFX96 Touch System. *BnRD21A* gene expression levels in each sample were normalized to that of the internal control rapeseed *actin* gene and are shown relative to the expression levels in the wild type. RT‐qPCR was performed with three biological replicates and three technical replicates. The gene‐specific primers used for RT‐qPCR are listed in Table S1.

### Yeast two‐hybrid and yeast three‐hybrid assay

For yeast two‐hybrid assay, OTU2 was cloned into pGBKT7 as bait, while RD21A without signal peptide (or other candidates) was cloned into pGADT7 as prey. Both plasmids were transformed into Y2H golden yeast cells using polyethylene glycol/LiAc‐mediated yeast transformation. The transformation mix was spread on SD medium−Leu−Trp (SD‐2, selects for both plasmids), and positive clones were further confirmed on SD medium−Trp−Leu−His−Ade (SD‐4) containing X‐α‐Gal and aureobasidin A. The experiment was repeated three times with similar results.

For yeast three‐hybrid assay, RD21A was cloned into the first multiple cloning site of the pBridge vector, while OTU2 was cloned into the second multiple cloning site of the pBridge vector or not (as a control), while PbE3‐2 without a signal peptide was cloned into pGADT7. PBridge and pGADT7 plasmids were transformed into Y2H golden yeast cells using polyethylene glycol/LiAc‐mediated yeast transformation. The transformation mix was spread on SD medium–Leu–Trp (selects for both plasmids), and positive clones were further confirmed on SD medium–Trp–Leu–His–Met containing X‐α‐Gal and aureobasidin A to evaluate the interaction between RD21A and PbE3‐2 in the presence or absence of OTU2. The experiment was repeated twice with similar results.

### Co‐IP assay

RD21A‐FLAG was co‐expressed with GFP‐OTU2 or GFP (as a control) in *N. benthamiana* by *Agrobacterium* infiltration. Eight *N. benthamiana* leaf discs with a diameter of 6 mm were ground and added to 350 μl IP buffer (20 mm Tris–HCl pH 7.5, 150 mm NaCl, 1% Triton X‐100, 5 mm EDTA, 1 mm PMSF and 1 × protease inhibitors) for cracking. The protein extracts were centrifuged at 12 000 ***g*** for 30 min. Taken 20 μl out of the sample for input detection, the rest of the supernatant was incubated with 5 μl α‐GFP agarose beads (gta‐20; ChromoTek, Germany) for 2 h at 4°C. After incubation, the beads were washed three times with IP buffer. The beads were pelleted, boiled in 20 μl 2 × SDS buffer and the proteins were separated by 12% SDS‐PAGE and detected by immunoblotting using α‐FLAG antibody to check whether RD21A‐FLAG was co‐immunoprecipitated. The experiment was repeated three times with similar results.

### Western blots assay

Proteins were separated by 10% SDS‐PAGE and transferred to 0.2‐μm PVDF membranes. The following antibodies were used: mouse anti‐FLAG (Sigma Aldrich) at a 1:2000 dilution, mouse anti‐GFP (ABclonal) at a 1:2000 dilution, rabbit anti‐HA (ABclonal) at a 1:2000 dilution, mouse anti‐GST (ABclonal) at a 1:2000 dilution, mouse anti‐Myc (Genscript) at a 1:2000 dilution, HRP‐conjugated anti‐rabbit IgG secondary antibodies (ABclonal) at a 1:10 000 dilution and HRP‐conjugated anti‐mouse IgG secondary antibodies (ABclonal) at a 1:10 000 dilution.

### Split luciferase complementation assays

The split‐LUC reconstitution assay was carried out as previously reported (Chen et al., 2008; Qi et al., 2022). OTU2 and RD21A were fused to N‐terminal luciferase (nLUC) and C‐terminal luciferase (cLUC), respectively. OTU2‐nLUC was co‐expressed with RD21A‐cLUC or vector control in *N. benthamiana* leaves via *Agrobacterium* infiltration (OD_600_ = 1.5). One micromolar luciferin (40901ES01; YEASEN, China) was infiltrated 48 h after infiltration, and luminescence was imaged within 5 min using a charge‐coupled device imaging apparatus. The experiment was repeated three times with similar results.

### BiFC assay

The BiFC assay was carried out as previously reported (Yang et al., 2018). OTU2 and RD21A were fused to the N‐terminus of RFP and the C‐terminus of RFP, respectively. *A. tumefaciens* GV3101 strains carrying the target plasmid are transiently expressed in the *N. benthamiana* and observed 48 h after *A. tumefaciens* GV3101 infiltration, detected with a confocal laser‐scanning microscopy (Nikon; A1HD25). RFP was detected after excitation with a 561 nm wavelength laser, and emissions were collected between 570 and 620 nm. Laser intensity was in the range of 2.0 to 3.0, and gain was in the range of 50 to 80. The experiment was repeated twice with similar results.

### Deubiquitination assay

For *in vivo* deubiquitination, Ub‐HA, RD21A‐FLAG and PbE3‐2‐GFP were co‐expressed with OTU2‐Myc or control Ev‐Myc in *N. benthamiana*, and total protein was extracted 2 days later with IP buffer (20 mm Tris–HCl pH 7.5, 150 mm NaCl, 1% Triton X‐100, 5 mm EDTA, 1 mm PMSF and 1 × protease inhibitors). IP was performed using anti‐HA beads, and ubiquitination and RD21A were detected by anti‐HA and anti‐FLAG antibodies, respectively. The experiment was repeated twice with similar results.

The *in vitro* deubiquitination assay was performed as previously described (Balakirev et al., 2003). The GST‐OTU2 recombinant protein or GST was first purified from *E. coli* strain BL21(DE3) for backup. RD21A‐Ub_n_, ubiquitinated by PbE3‐2, was further purified according to the previous method (Han et al., 2017). Two milliliters of RD21A‐Ubn protein were purified from 100 ml DE3 strain. After ultrafiltration (150 mm NaCl, 20 mm Tris–HCl, pH 8), 50 μl RD21A‐Ubn protein was mixed with 10 μg OTU2‐GST or 10 μg GST in the reaction buffer (150 mm NaCl, 20 mm Tris–HCl, pH 8). RD21A‐Ubn and OTU2‐GST or GST were detected by western blot after reaction at 37°C for 1 h. The experiment was repeated three times with similar results.

## ACCESSION NUMBERS

Sequence data from this article can be found in the GenBank/EMBL data libraries under accession numbers OTU2, NP_001321195.1; RD21A, NP_564497.1; BnRD21A, CDY17044.1; PbE3‐2, ON394061, respectively.

## AUTHOR CONTRIBUTIONS

CL and TC conceived the project and designed experiments. CL, SL and LF performed experiments and analyzed the data. JC, JX, YL, YF, KT and DJ analyzed the data and provided advice that helped shape the research. CL and TC wrote the manuscript with input from all coauthors.

## CONFLICT OF INTEREST

The authors declare no conflicts of interest.

## Supporting information


**Figure S1.** Western blot to identify the RD21A/PbE3‐2 overexpression plants and to detect protein expression in Y2H and BiFC assays. (a) α‐FLAG and α‐GFP antibodies were used to detect RD21A‐FLAG and PbE3‐2‐GFP, respectively. Protein loading was shown by Ponceau S staining of Rubisco. (b) Y2H assays show that OTU2 interacts with RD21A, iRD21A and mRD21A but not with PbE3‐2. Immunoblot analysis was performed using α‐HA and α‐MYC antibodies to detect the accumulation of OTU2 and the candidate interacting proteins. (c) BiFC confirmed the interaction between OTU2 and RD21A. OTU2 is fused to the N‐terminus of RFP; RD21A is fused to the C‐terminus of RFP. The corresponding GV3101 strains carrying the target plasmid are transiently expressed in the *N. benthamiana*. Immunoblot with α‐MYC or α‐FLAG antibody was performed to detect protein accumulation. The experiment was repeated twice with similar results.
**Figure S2.** RD21A interacts with truncated OTU2. (a) The evolutionary relationships of the *A. thaliana* OTU family genes were determined with the neighbor‐joining algorithm. (b) Protein alignment of OTU2 and OTU8. (c) Schematic representation of functional motifs present in OTU2, OTU2 indicated the full length, OTU2n indicated N‐terminal (11–123 aa) and OTU2c indicated C‐terminal (124–208 aa). (d) Y2H assay showed that RD21A interacts with OTU2c. The experiment was repeated three times with similar results.
**Figure S3.** OTU2 inhibits the degradation of RD21A by PbE3‐2 and MiCE108. (a) *Agrobacterium* harboring RD21A‐FLAG, PbE3‐2‐GFP and OTU2‐Myc or Ev‐Myc constructs were coinfiltrated in *N. benthamiana* leaves. Immunoblotting was performed with α‐FLAG, α‐GFP and α‐Myc antibodies against RD21A‐FLAG, PbE3‐2‐GFP and OTU2‐Myc, respectively. (b) RD21A‐FLAG and MiCE108‐GFP were co‐expressed with OTU2‐MYC or Ev‐MYC (as a control) in *N*. *benthamiana* leaf by *Agrobacterium* infiltration. Total protein was extracted with IP buffer (P0013; Beyotime, China) after 2 days. Immunoblot with α‐FLAG antibody was performed to detect RD21A‐FLAG protein accumulation. Protein loading was shown by Ponceau S staining of Rubisco. The experiment was repeated twice with similar results.
**Figure S4.** Growth phenotype of Col‐0, *OTU2* overexpression and *otu2* mutant plants. (a) Phenotype of T2 generation of 4‐week‐old *OTU2* overexpression and *otu2* mutant plants. (b) Validation of *otu2* T‐DNA insertion mutants. PCR assays were performed with LP + RP primers to detect OTU2 and LB + RP primers to detect T‐DNA. DNA from Col‐0 and *otu2* plants was used as templates. (c) Validation of *OTU2* transcription levels. (d) Western Blot to identify the T2 generation of OTU2 overexpression lines with α‐Myc antibody (top). Protein loading was shown by Ponceau S staining of Rubisco (bottom). (e) RD21A protein level in *OTU2* overexpression and *otu2* mutant *A. thaliana*. Total protein was extracted from three leaves of three plants. Immunoblotting was performed with α‐RD21A antibody (top). Protein loading was shown by Ponceau S staining of Rubisco (bottom). The experiment was repeated three times with similar results.
**Figure S5.** Western blot to identify the OTU2‐FLAG overexpression plants and to detect RD21A accumulation during *P. brassicae* infection. (a) Western blot to identify the T2 generation of *OTU2* overexpression lines in *rd21a* or Col‐0 background with α‐FLAG antibody (top). Protein loading was shown by Ponceau S staining for Rubisco (bottom). (b) OTU2 overexpression Arabidopsis line accumulates higher RD21A protein levels during *P. brassicae* infection. Total protein was isolated from the roots of 20 Arabidopsis seedlings which were 16‐day‐old and grown on half‐strength MS medium, and infected with *P. brassicae* for 2 days. Immunoblot with α‐RD21A antibody was performed to detect RD21A protein accumulation. The experiment was repeated three times with similar results.
**Figure S6.** Working model for OTU2 to regulate *A. thaliana* resistance to *P. brassicae*. *P. brassicae* secretes PbE3‐2, which ubiquitinates and degrades RD21A to suppress the *A. thaliana* immune response. In response, *A. thaliana* deubiquitinating enzyme OTU2 inhibits the interaction between RD21A and PbE3‐2 and increases the stability of RD21A by deubiquitinating it thereby increasing resistance to *P. brassicae*.

**Table S1.** Primers used in the study.
**Table S2.** PbE3‐2‐associated deubiquitinating enzymes in IP‐MS/MS analysis.

## Data Availability

The data that supports the findings of this study are available in the supplementary material of this article.
