# Supplementary material for: Arabidopsis OTU2 deubiquitinates cysteine protease RD21A to enhance clubroot resistance
Source: Plant J. 2025 Apr 14;122(1):e70148. doi: 10.1111/tpj.70148 (PMC11995443; doi:10.1111/tpj.70148)
Supplement: Supplementary file 1 — Figure S1. Western blot to identify the RD21A/PbE3‐2 overexpression plants and to detect protein expression in Y2H and BiFC assays. (a) α‐FLAG and α‐GFP antibodies were used to detect RD21A‐FLAG and PbE3‐2‐GFP, respectively. Protein loading was shown by Ponceau S staining of Rubisco. (b) Y2H assays show that OTU2 interacts with RD21A, iRD21A and mRD21A but not with PbE3‐2. Immunoblot analysis was performed using α‐HA and α‐MYC antibodies to detect the accumulation of OTU2 and the candidate interacting proteins. (c) BiFC confirmed the interaction between OTU2 and RD21A. OTU2 is fused to the N‐terminus of RFP; RD21A is fused to the C‐terminus of RFP. The corresponding GV3101 strains carrying the target plasmid are transiently expressed in the N. benthamiana. Immunoblot with α‐MYC or α‐FLAG antibody was performed to detect protein accumulation. The experiment was repeated twice with similar results. Figure S2. RD21A interacts with truncated OTU2. (a) The evolutionary relationships of the A. thaliana OTU family genes were determined with the neighbor‐joining algorithm. (b) Protein alignment of OTU2 and OTU8. (c) Schematic representation of functional motifs present in OTU2, OTU2 indicated the full length, OTU2n indicated N‐terminal (11–123 aa) and OTU2c indicated C‐terminal (124–208 aa). (d) Y2H assay showed that RD21A interacts with OTU2c. The experiment was repeated three times with similar results. Figure S3. OTU2 inhibits the degradation of RD21A by PbE3‐2 and MiCE108. (a) Agrobacterium harboring RD21A‐FLAG, PbE3‐2‐GFP and OTU2‐Myc or Ev‐Myc constructs were coinfiltrated in N. benthamiana leaves. Immunoblotting was performed with α‐FLAG, α‐GFP and α‐Myc antibodies against RD21A‐FLAG, PbE3‐2‐GFP and OTU2‐Myc, respectively. (b) RD21A‐FLAG and MiCE108‐GFP were co‐expressed with OTU2‐MYC or Ev‐MYC (as a control) in N. benthamiana leaf by Agrobacterium infiltration. Total protein was extracted with IP buffer (P0013; Beyotime, China) after 2 days. Immunoblot with α‐FLAG a [file TPJ-122-0-s001.pdf]

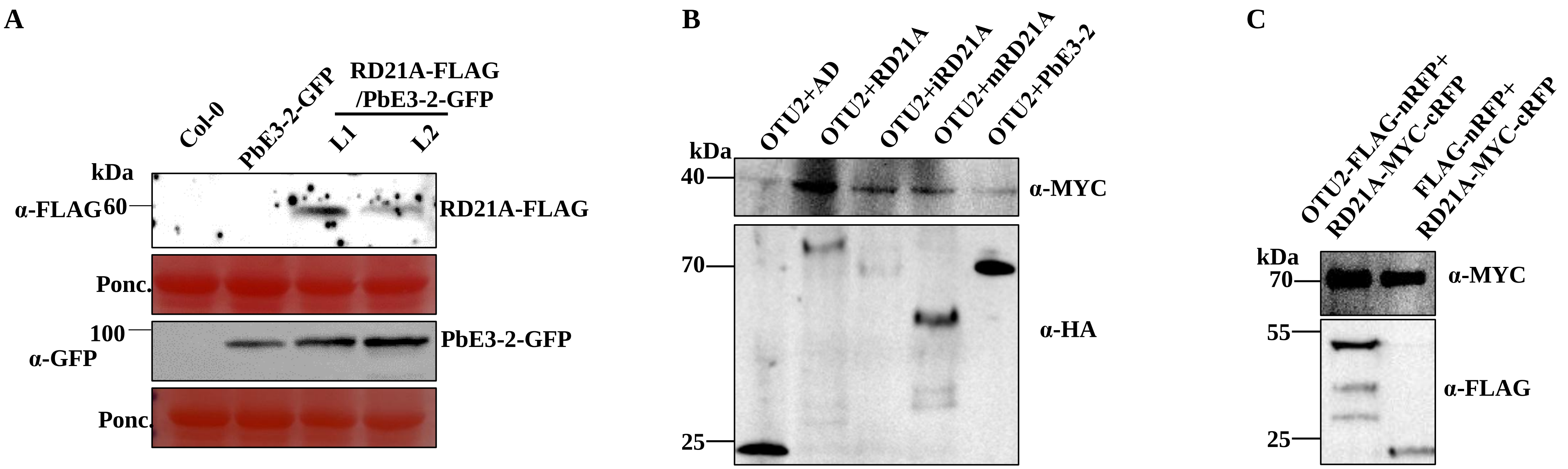

Figure S1. Western Blot to identify the RD21A/PbE3-2 overexpression plants and to detect protein expression in Y2H and BiFC assays.

(A)α-FLAG and α-GFP antibodies were used to detect RD21A-FLAG and PbE3-2-GFP, respectively. Protein loading was shown by Ponceau S staining of Rubisco. (B) Y2H assays show that OTU2 interacts with RD21A, iRD21A and mRD21A but not with PbE3-2. Immunoblot analysis was performed using α-HA and α-MYC antibodies to detect the accumulation of OTU2 and the candidate interacting proteins. (C) BiFC confirmed the interaction between OTU2 and RD21A. OTU2 is fused to the N-terminus of RFP; RD21A is fused to the C-terminus of RFP. The corresponding GV3101 strains carrying the target plasmid are transiently expressed in the *N. benthamiana*. Immunoblot with α-MYC or α-FLAG antibody was performed to detect protein accumulation. The experiment was repeated twice with similar results.

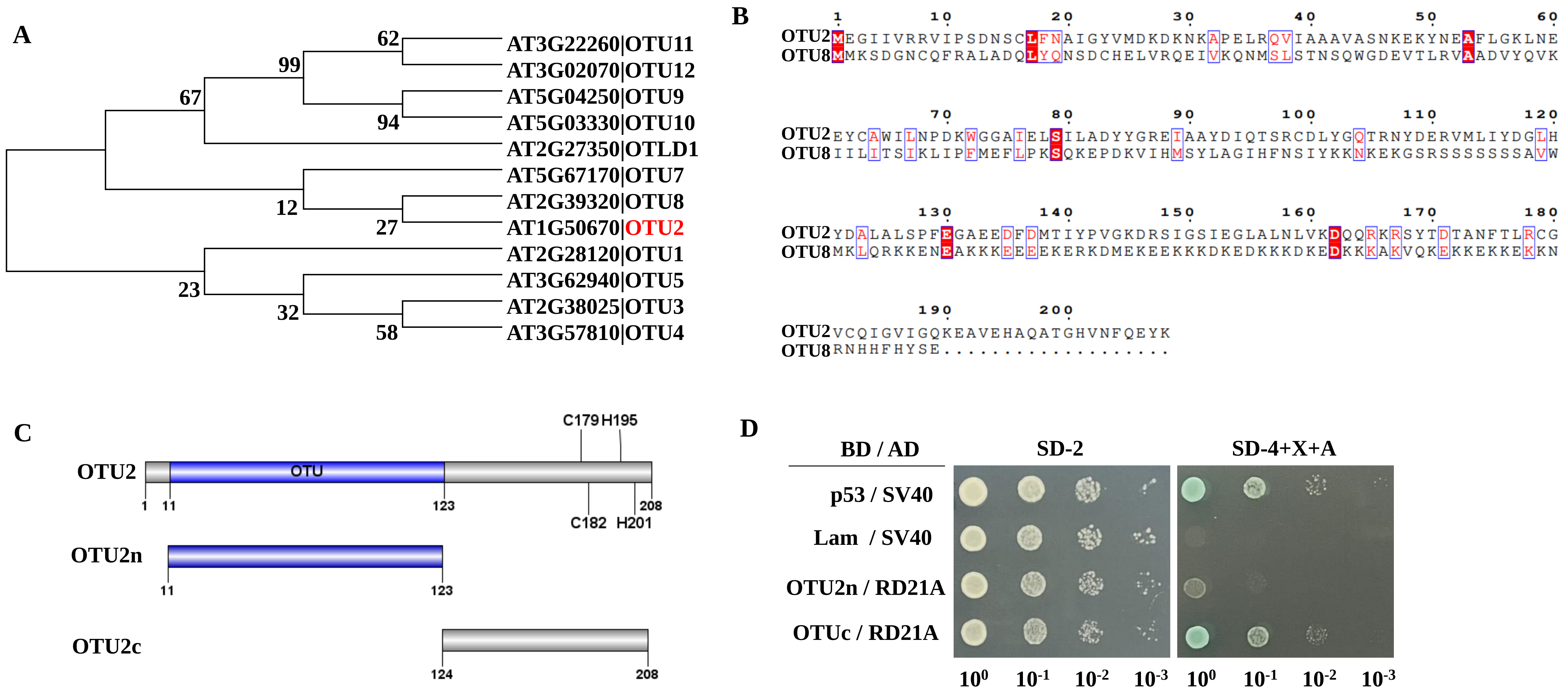

Figure S2. RD21A interacts with truncated OTU2.

(A) The evolutionary relationships of the *A. thaliana* OTU family genes were determined with the neighbor-joining algorithm. (B) Protein alignment of OTU2 and OTU8. (C) Schematic representation of functional motifs presents in OTU2, OTU2 indicated the full length, OTU2n indicated N-terminal (11-123 aa) and OTU2c indicated C-terminal (124-208 aa). (D) Y2H assay showed that RD21A interacts with OTU2c. The experiment was repeated three times with similar results.

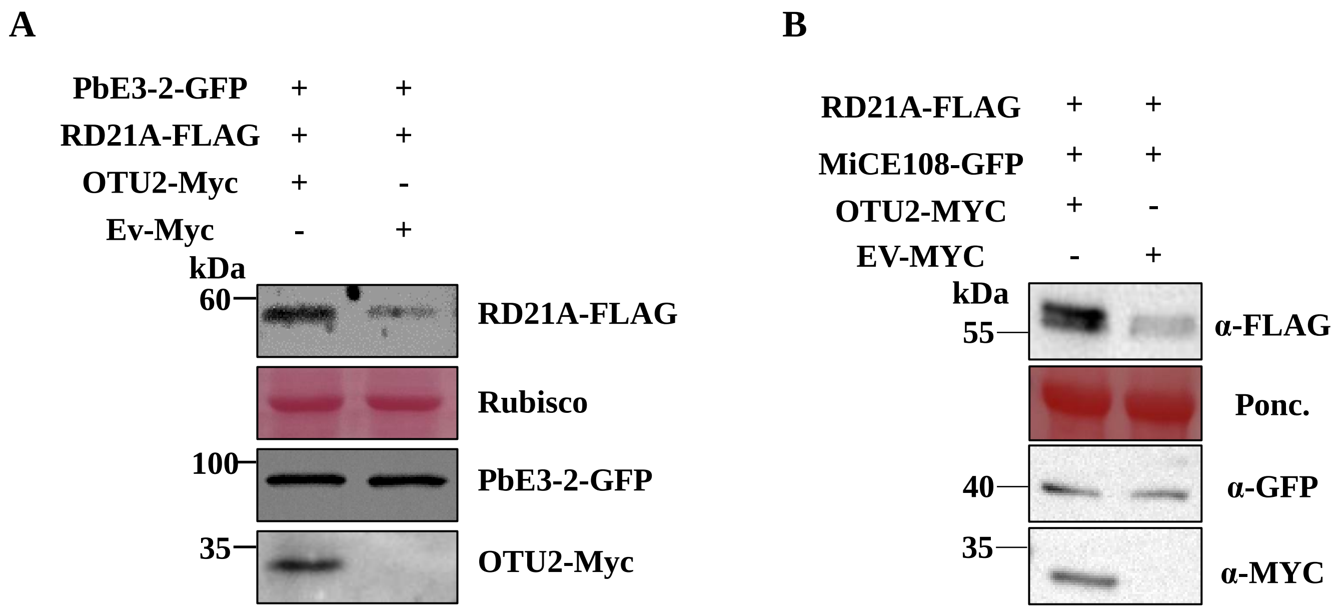

Figure S3. OTU2 inhibits the degradation of RD21A by PbE3-2 and MiCE108.

(A) *Agrobacterium* harboring RD21A-FLAG, PbE3-2-GFP and OTU2-Myc or Ev-Myc constructs were co-infiltrated in *N. benthamiana* leaves. Immunoblotting was performed with α-FLAG, α-GFP and α-Myc antibodies against RD21A-FLAG, PbE3-2-GFP and OTU2-Myc, respectively. (B) RD21A-FLAG, MiCE108-GFP were co-expressed with OTU2-MYC or Ev-MYC (as a control) in *N. benthamiana* leaf by *Agrobacterium* infiltration. Total protein was extracted with IP buffer (Beyotime, P0013, China) after 2 days. Immunoblot with α-FLAG antibody was performed to detect RD21A-FLAG protein accumulation. Protein loading was shown by Ponceau S staining of Rubisco. The experiment was repeated twice with similar results.

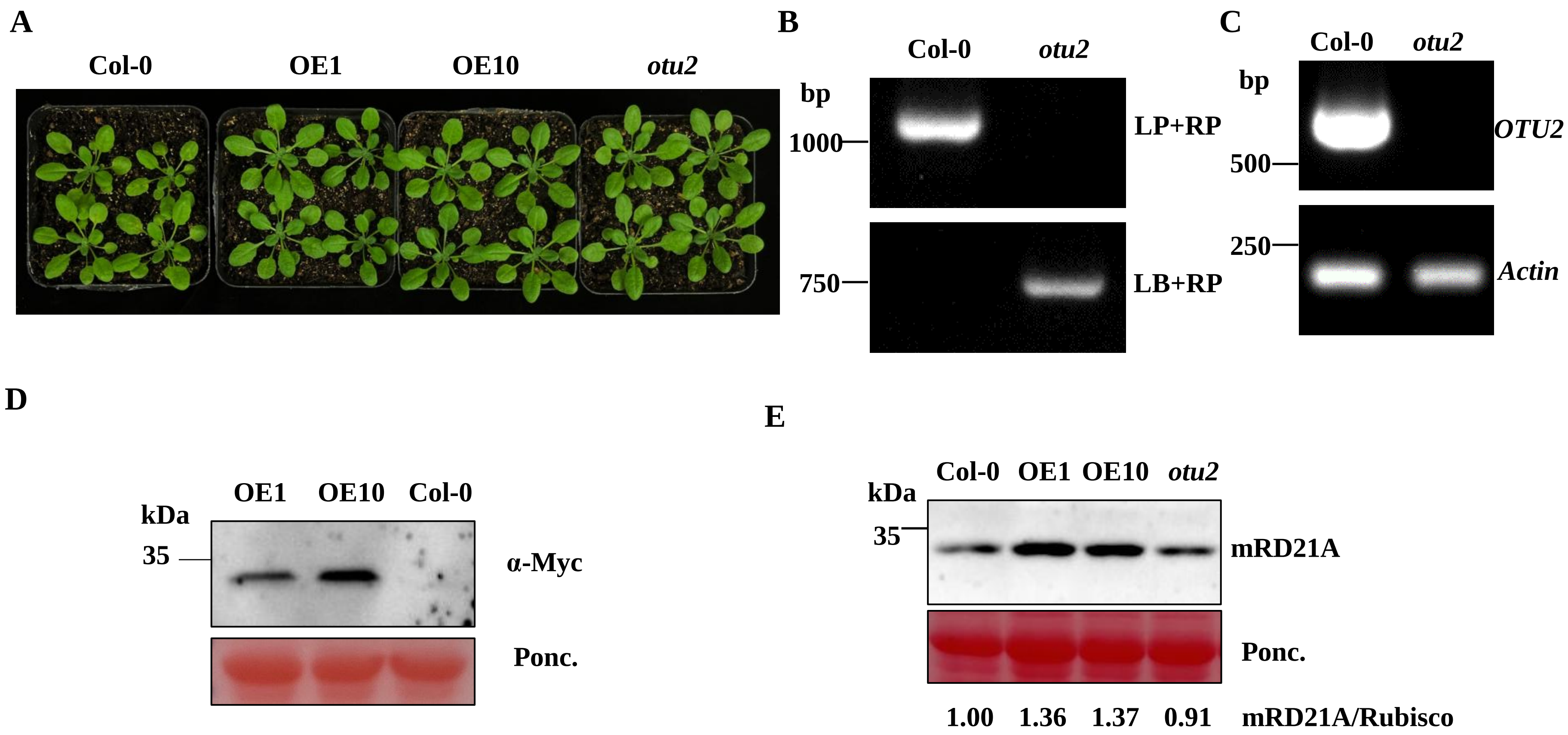

Figure S4. Growth phenotype of Col-0, ***OTU2*** **overexpression and** ***otu2*** **mutant plants.**

(A) Phenotype of T2 generation of 4-week-old *OTU2* overexpression and *otu2* mutant plants. (B) Validation of *otu2* T-DNA insertion mutants. PCR assays were performed with LP+RP primers to detect OTU2, and LB+RP primers to detect T-DNA. DNA from Col-0 and *otu2* plants were used as templates. (C) Validation of *OTU2* transcription levels. (D) Western Blot to identify the T2 generation of OTU2 overexpression lines with α-Myc antibody (top). Protein loading was shown by Ponceau S staining of Rubisco (bottom). (E) RD21A protein level in *OTU2* overexpression and *otu2* mutant *A. thaliana*. Total protein was extracted from three leaves of three plants. Immunoblotting was performed with α-RD21A antibody (top). Protein loading was shown by Ponceau S staining of Rubisco (bottom). The experiment was repeated three times with similar results.

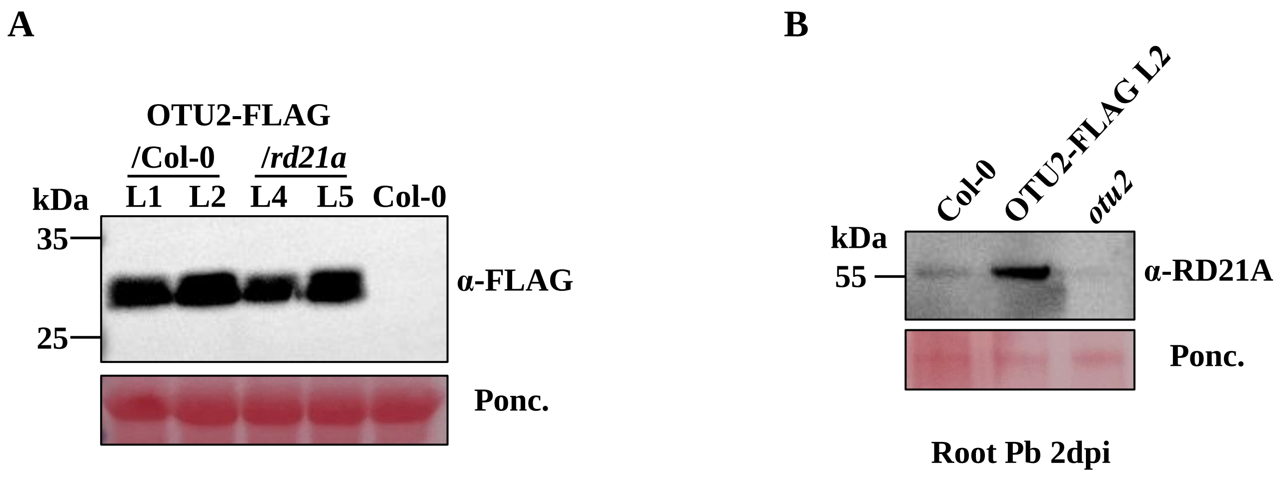

Figure S5. Western Blot to identify the OTU2-FLAG overexpression plants and to detect RD21A accumulation during *P. brassicae* infection.

(A) Western Blot to identify the T2 generation of *OTU2* overexpression lines in *rd21a* or Col-0 background with α-FLAG antibody (top). Protein loading was shown by Ponceau S staining for Rubisco (bottom). (B) OTU2 overexpression *Arabidopsis* line accumulates higher RD21A protein levels during *P. brassicae* infection. Total protein was isolated from the roots of 20 *Arabidopsis* seedlings which were 16-day-old and grown on half-strength MS medium, and infected with *P. brassicae* for 2 days. Immunoblot with α-RD21A antibody was performed to detect RD21A protein accumulation. The experiment was repeated three times with similar results.

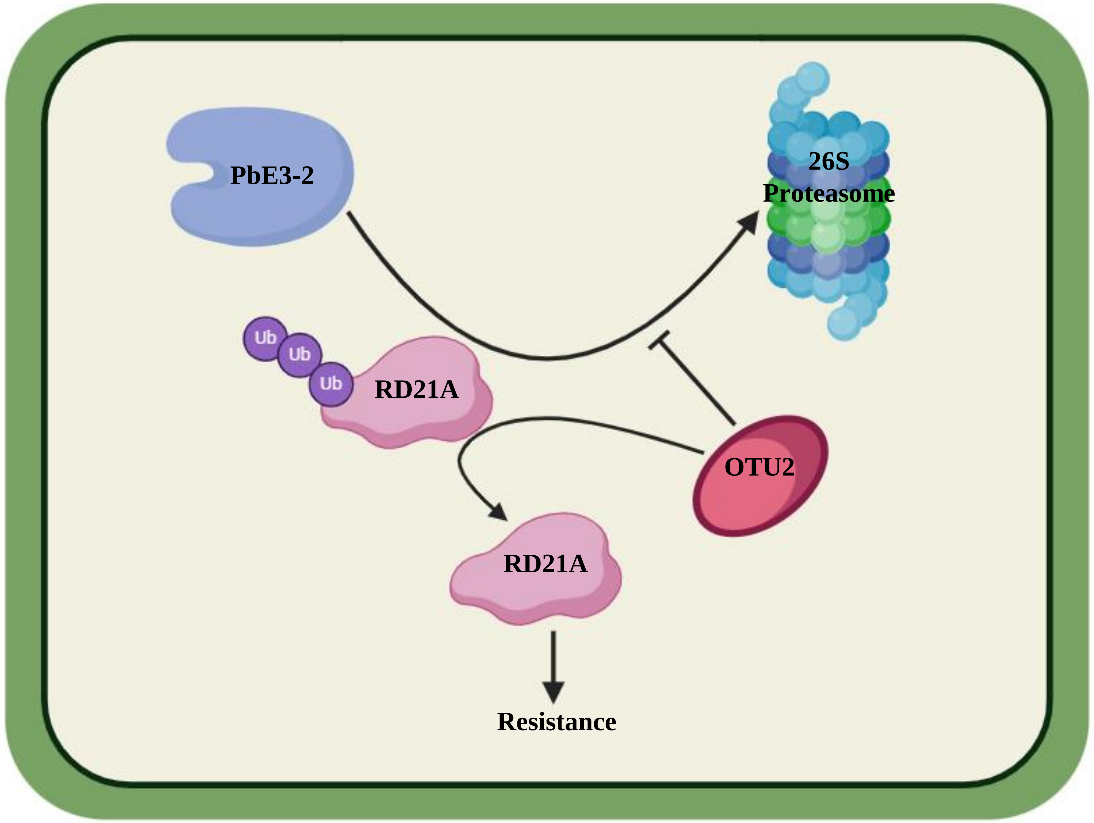

Figure S6. Working model for OTU2 to regulate *A. thaliana* resistance to *P. brassicae.*

P. brassicae secretes PbE3-2, which ubiquitinates and degrades RD21A to suppress *A. thaliana* immune response. In response, *A. thaliana* deubiquitinating enzyme OTU2 inhibits the interaction between RD21A and PbE3-2, and increases the stability of RD21A by deubiquitinating it thereby increasing resistance to *P. brassicae.*
